# American Dental Asssociation

**Published:** 1895-12

**Authors:** 


### American Dental Association.

We here present an abstract of the remarks of discussion on the papers of Drs. Stainton,* Jack, and Barrett:

Dr. Thos. Fillebrown said there was a general impression that the Faculties Association could prevent the establishment of new dental schools, but this is an error, for they have no power to prohibit them. He thought the number of physicians given in the report was underestimated. That, instead of there being already too many dentists, more were needed, and the graduates, from year to year, did not supply the normal increase. The holder of a diploma looks upon it as a great hardship to be required to pass an examination before an examining board, before being permitted to practice in some of the other States; but that is all right. In Massachusetts they have a law of this kind for lawyers and he thought physicians as well as dentists. It seems to be a good whip to hold over the schools, and he would like to see this requirement in every State.

Dr. Jas. McManus spoke of a bill being passed in the Legislature of Connecticut, to incorporate a dental college in Connecticut. The Board of Examiners knew nothing of the movement and there were not twenty dentists in the whole State that wanted such a college, for there was no demand for it. The State Society took the matter in hand and succeeded in getting the Senate to defeat the measure. They did not want a dental school incorporated that in the nature of things could not be a success.

Dr. C. S. Stockton asked if it was not possible for the colleges to turn out graduates that were gentlemen and reputable dentists? So many all over the country are advertising, and it is a great detriment to the profession. He thought that the colleges could remedy this evil to a certain extent by giving the graduate to understand that if he practiced unprofessionally his diploma would be recalled.

Dr. M. L. Rhein spoke about the enormous amount of poor timber there is among men who call themselves dentists. How

*See Dr. Stainton's article Nov. No. page 536.

are we going to elevate the character of the profession by putting a Chinese wall about us and endeavoring to keep out practitioners, as has been done by a great many States in this country by the laws that have been enacted, and some of which will not bear judicial inquiry? He denied the idea of there being already too many dentists to meet the demand. Even if there were no increase in our pupulation, he believed that the enlightenment of the people alone would keep up a demand for all the dentists that the colleges turn out. It would be a great blot on this association to let it go out to the masses that they had taken the position advocated in some of the papers. The vital question is, can we make better dentists out of the mass of men already in our profession? The administration of an oath will not help matters. What we need is the establishment of better institutions than we have to-day.

Dr. J. Foster Flagg said that dentistry with him was first, greatest, best all the time. He referred to Dr. Jack's speaking of a great danger threatening our profession. Are we a profession? Are we united in regarding ourselves as a profession? A few minutes after that he, Jack, spoke of the great facilities which universities have to educate men for our specialty. Dr. Flagg said we are all striving to better our profession, and he believed that the university was the very thing to educate men for the profession, but not through the medical physiologist, anatomist, chemist, etc. Regarding State boards, he said they were the foundation for the progress of dentistry, and for a man to be examined from State to State appeared to be the proper thing.

Dr. C. P. Lennox thought the difficulty was more with the people than with the schools. We have for years talked about educating the people, but it is a difficult thing to do. He believed that dentists should be thoroughly educated themselves, but this alone will not remedy the evil of cheap dentistry. The only way to overcome it is to educate the masses.

Dr. E. A. Bogue said that he agreed mainly with what Dr. Flagg had said. It is in vain for us to apply to the colleges to recall their diplomas after they have been given out, unless there is a great moral force brought to bear on the subject. He thought that between the National Association of Examiners and Faculties, together with the moral weight of the American Association, this question could be settled. If there is formulated one standard of education then the States can properly accept diplomas, one from the other.

Dr. J. N. Crouse thought that colleges should not charge a fee for clinical work. This would, to a certain extent, have its influence, for many of the colleges being started are run for the money that can be made out of them.

Dr. Louis Jack said that the purpose of the second paper was to make apparent the necessity for care in the admission of new schools. This is necessary to bring about a higher standard of dental education than now exists. He spoke about the plan of requirements for the recognition of dental schools as adopted by the National Board of Examiners. (See proceedings in the October issue of this journal page 504.)

These requirements will not only bring up the standard of the new schools, but will influence the old ones as well.

Dr. L. D. Shepard said that Dr. Crouse had presented the only solution to the problem. The general impression was that the colleges charged only the actual cost of material for the work done in the infirmary. This was absolutely a falsehood. If the infirmary is a source of great revenue, and he had been informed by good authority that in some instances it netted thousands of dollars, then the solution offered by Dr. Crouse was an admirable one. He spoke of the idea that the dental profession was a liberal one, and yet there was not one dental hospital where the poor could get dental service for nothing. How many are giving a half day a week to the service of the poor? With the medical men it was different. They had their free hospitals, and gave their services free. What to do for the teeth of the poor was a vital question, and he hoped to see the day when these hospitals would be established.

Dr. James Truman said: There is not a year that somebody does not assail the colleges. When a man stands up before this great representative body of dentists and says that there is not a dental college in the land that is not a fraud, it is time to stop and consider what we are doing. The charge that the dental colleges are making large sums of money annually out of their infirmary practice, which is supposed to be largely gratuitous, is not true—at least it is not true of the school with which the speaker is connected, and, he believed, not true of the majority of them. They are doing a great service for the poor in caring for their teeth at the lowest possible cost. It is a disgrace to this body that its members will sit and listen to such charges, made in a general way, against the institutions which have been building up dentistry for forty years. There is one thing which must be borne in mind in connection with this matter of caring for the teeth of the poor, and that is that you cannot get at the very poor. The exigencies of their lives are such that they have not the time to come to the infirmary—cannot spare it from the necessities of earning their living. Those who do come must frequently wait for three or four hours to be served. Again, there can never be any comparison of the dentist with the medical man in the matter of gratuitous service to the poor, because of the total dissimilarity of the services which each has to perform.

Dr. H. A. Smith said that the institution with which he was connected did have a hospital in which it was doing gratuitous work. They treated the teeth of children in charitable institutions, and gave them not only free service but made no charge for material used. He mentioned this merely to show that there were some of the colleges doing charitable work, and the charges made that they were not was untrue.

Dr. W. W. Walker thought there was room for more colleges if the standard was raised high enough.

